# A case of giant intradiaphragmatic bronchogenic cyst

**DOI:** 10.1002/rcr2.832

**Published:** 2021-08-17

**Authors:** Naoya Kitamura, Tomohiko Takahashi, Tetsuya Takayama, Jun Kawamukai, Hideki Shinno, Hideki Miyazawa

**Affiliations:** ^1^ Department of Thoracic Surgery Toyama Prefectural Central Hospital Toyama Japan

**Keywords:** bronchogenic cysts, congenital anomaly, extensive resection, intradiaphragmatic, thoracotomy

## Abstract

We present the case of a giant bronchogenic cyst (BC) that appeared just within the right diaphragm. A 51‐year‐old man was referred to our hospital with a chief complaint of pain from the lumbar area to the right shoulder. Computed tomography images showed a cystic mass measuring 18.0 × 17.5 × 12.8 cm in the right thoracic cavity. Right posterolateral thoracotomy from the eighth intercostal space was performed, and the cyst wall and diaphragm were resected together. The defect of the diaphragm was repaired using a 2‐mm‐thick Gore‐Tex™ expanded polytetrafluoroethylene patch. It is embryologically rare for a giant BC to develop within the right diaphragm. As BCs may be associated with malignant tumours or infection, complete resection of the cyst wall is required. Literature review revealed no consensus on the best surgical procedure. Therefore, it is important to consider the appropriate surgical procedure for each case.

## Graphical abstract

We present an exceedingly rare case of a giant bronchogenic cyst that had developed within the right diaphragm. The cyst was successfully surgically resected.

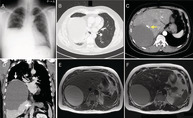

## INTRODUCTION

Bronchogenic cysts (BC)s are congenital lesions that occur during the early embryonic development and generally appear within the mediastinum and lung parenchyma. However, it is an exceedingly rare case that develops just within the diaphragm. Herein, we present a surgically resected case of a giant BC developed within the right diaphragm.

## CASE REPORT

**FIGURE 1:**
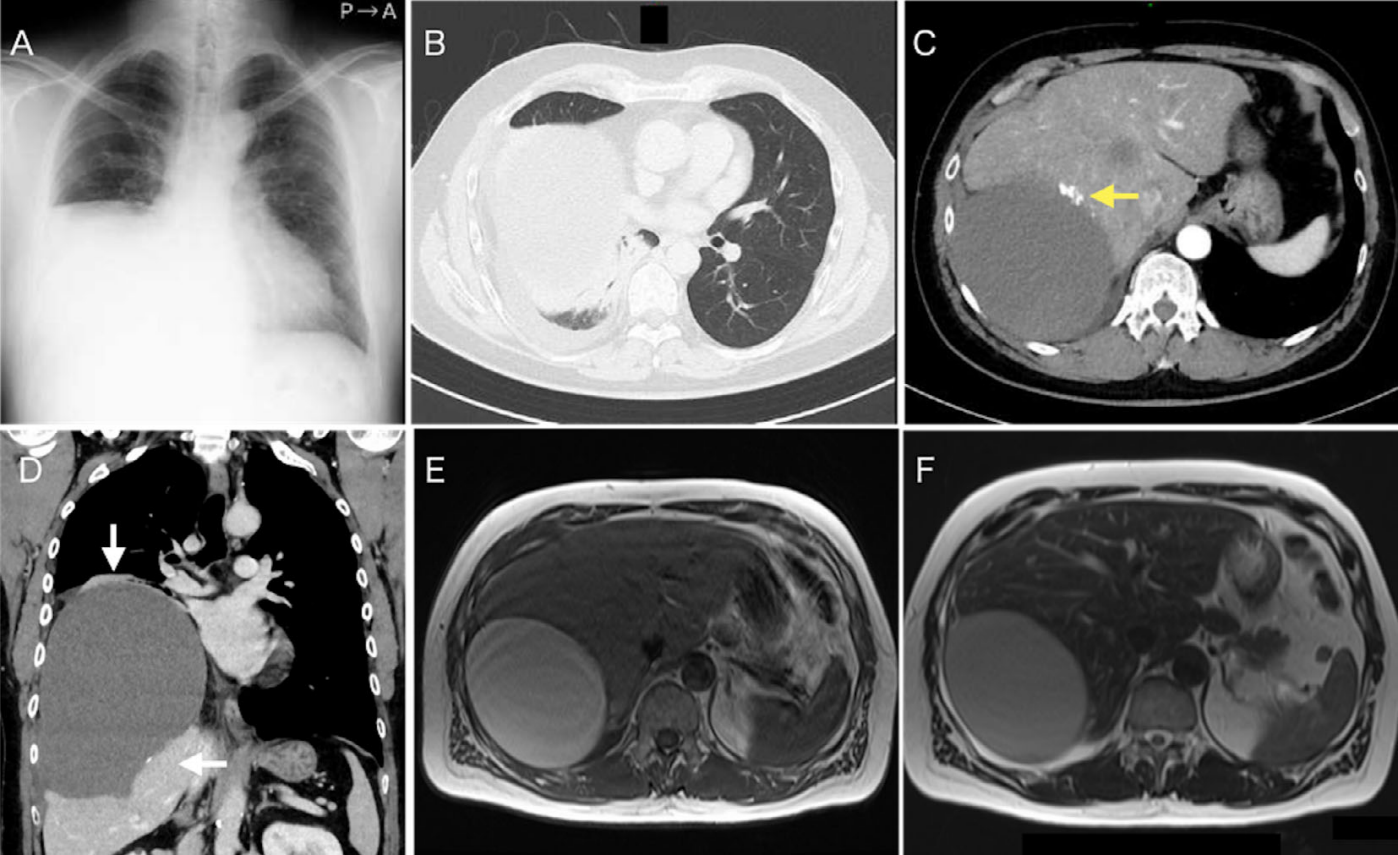
(A) Chest x‐ray shows decreased radiopacity of the right lower lung field. (B–D) Enhanced chest computed tomography images show a cystic mass with no contrast enhancement measuring 18 × 17.5 × 12.8 cm in the right thoracic cavity. Mass effects of the lower lobe of the right lung and liver (white arrow) and partial calcification (yellow arrow) are also revealed. (E, F) Chest magnetic resonance imaging suggests a cystic tumour, but it was difficult to identify until the origin could be determined

**FIGURE 2:**
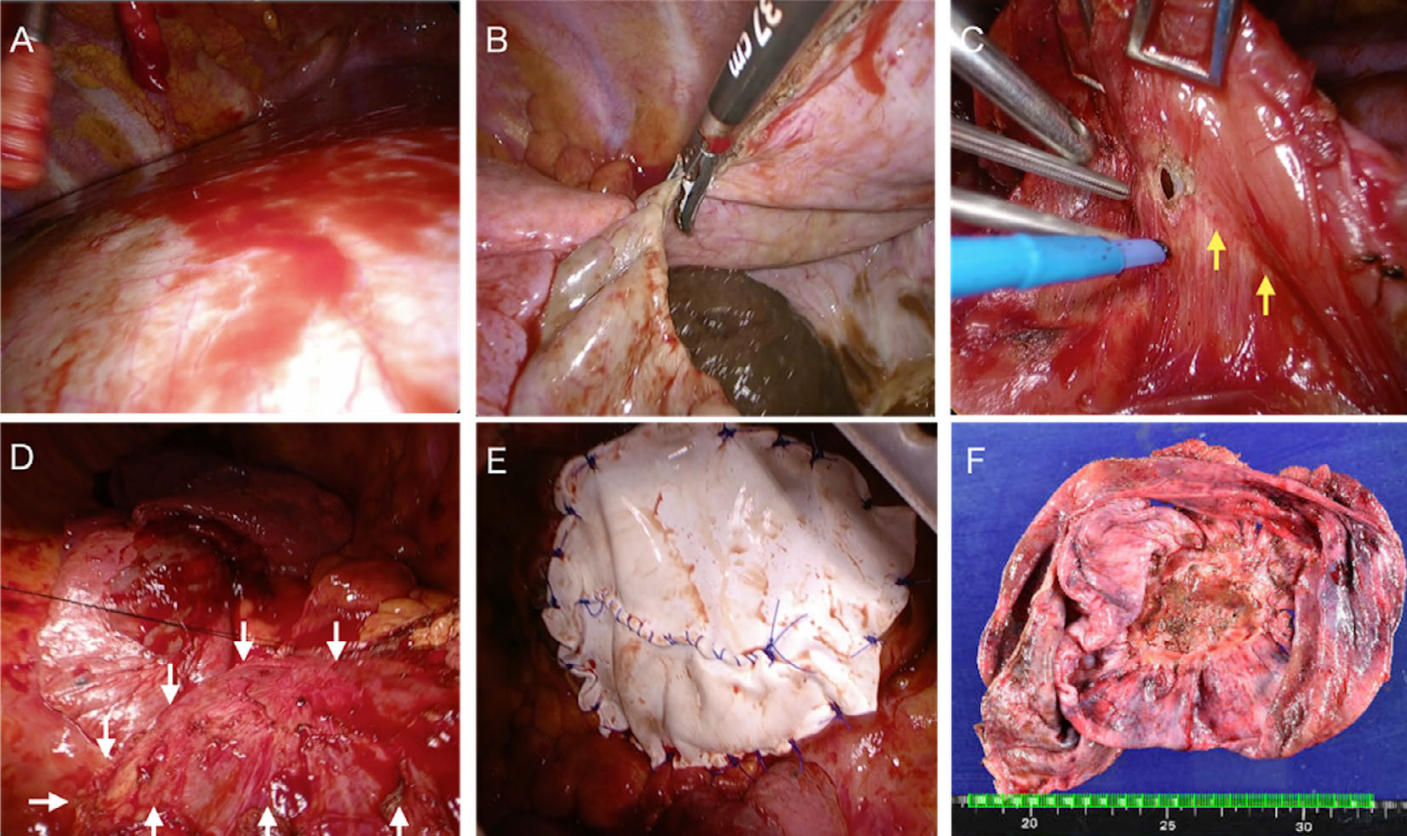
(A) The boundary of the cyst was unclear from the diaphragmatic surface, suggesting intramuscular development. (B) When the diaphragm was incised using the vessel sealing system, brown debris was found in the cyst. (C, D) Muscle layers of the diaphragm and the cyst wall were strongly adhered (yellow arrows), which were resected together (white arrows). (E) The defect of the diaphragm was repaired using Gore‐Tex™ expanded polytetrafluoroethylene patch to prevent diaphragmatic hernia. (F) Histologically, it was a single tufted cyst measuring 14.5 × 14.5 cm, and no obvious malignant findings were observed

A 51‐year‐old man was referred to our hospital with a complaint of pain spreading from the lumbar area to the right shoulder. A chest x‐ray showed decreased radiopacity in the right lower lung field (Figure 1A). Although a physician had noted an abnormal opacity on a chest x‐ray 3 years ago, further examination of the opacity was not performed because the patient was asymptomatic. Computed tomography images showed a non‐solid mass measuring 18 × 17.5 × 12.8 cm in the right thoracic cavity (Figure 1B–D). The mass was accompanied by compression of the lower lobe of the right lung and the liver. Chest magnetic resonance imaging (MRI) suggested a cystic tumour derived from the pleura or diaphragm (Figure 1E,F). Blood tests showed no increase in inflammatory marker, but a high cancer antigen 19‐9 (CA 19‐9) level was observed (11,726 U/ml). The cyst fluid culture was negative, and the cytology showed no evidence of malignancy. The patient's pain was accompanied by shortness of breath, and we judged that surgery was indicated for diagnostic treatment. We first attempted to remove the cyst wall through thoracoscopic surgery, but a large cyst was identified in the diaphragm, and extensive resection was considered necessary. Therefore, we performed posterior lateral thoracotomy from the eighth intercostal space. The diaphragm was incised and brown fluid with a foul smell was yielded. The cyst wall together with the densely adhered diaphragmatic muscle layers were resected. The defect of the diaphragm measuring about 12 × 10 cm was repaired using a 2‐mm‐thick Gore‐Tex™ expanded polytetrafluoroethylene (ePTFE) patch (W. L Gore & Associates, Delaware, United States). The patient was discharged on the eighth post‐operative day. His respiratory function and shortness of breath improved 1 month after surgery (forced vital capacity [FVC]: 2.32 – 2.61 L, forced expiratory volume in 1 s [FEV1]: 1.66 – 2.05 L, FEV1/FVC: 74.4 – 82.7%). Pathological diagnosis was a BC with partial calcification lined by stratified squamous epithelium. No malignant findings were observed in the cyst wall or cyst fluid. Ten months have passed since the operation; there has been no recurrence and the level of CA 19‐9 has normalized (Figure 2). The diaphragm was incised and brown fluid with a foul smell was yielded (Fig. 2A, B). The cyst wall together with the densely adhered diaphragmatic muscle layers were resected. The defect of the diaphragm measuring about 12 × 10 cm was repaired using a 2 mm thick Gore‐Tex™ expanded Polytetrafluoroethylene (ePTFE) patch (W. L. Gore & Associates, Delaware, United States) (Fig. 2C, D, E, F).

## DISCUSSION

BCs are congenital anomalies that occur during the embryonic period due to abnormal germination or invasion of the foregut. The cysts occur most often in the posterior mediastinum and may occur ectopically in the neck or retroperitoneum; however, they are extremely rare in the diaphragm.^1^ Diaphragmatic BCs are characterized by (1) predominantly on the left (71.4%), (2) in women (57%) and (3) in the vertebro‐phrenic angle (70%).^1^, ^2^ The average size has been reported as 6.19 ± 2.58 cm, with the maximum reported size as 10 cm.^2^ Based on the above‐mentioned facts, it is exceedingly rare for a BC with a diameter of 18 cm (histologically 14.5 cm) to develop within the right diaphragm as in this case. However, there were no obvious infection or malignant findings, and the cause of growth was unclear.

MRI is useful as an adjunct to diagnosis and is recommended to exclude teratomas and neoplastic cystic lesions.^3^ In addition, it is known that the level of CA 19‐9 is elevated in BCs as in this case, and it has been suggested that CA 19‐9 may be useful in the diagnosis.^4^ However, histopathological assessment is the only approach to achieve a definitive diagnosis. In this case, cyst aspiration was performed for the purpose of diagnosis, but it may be unnecessary, considering the risk of infection. Complete resection of the cyst wall is indicated for both diagnostic and therapeutic purposes because of the possibility of malignancy and risk of secondary infections.^2^, ^3^

The most common surgical approach is posterior lateral thoracotomy (47.4%) and the most common reconstructive method is primary closure (69.2%).^2^ While there are reports that thoracoscopic resection is possible, there are also reports of using Gore‐Tex™ ePTFE patch to repair the diaphragmatic defect, suggesting that it is necessary to select a surgical method and approach specific for each case. In this case, we first attempted to remove the cyst wall through thoracoscopic surgery. However, the cyst was large and strong adhesions to the diaphragmatic muscularis could have resulted in incomplete resection of the cyst wall. Therefore, diaphragmatic resection was performed under thoracotomy to ensure curability, and diaphragm reconstruction was performed using a patch to prevent diaphragmatic hernia. The approach and repair method are defined by the size, location of the cyst (diaphragm surface, inside, edge, centre, etc.) and the modes of diaphragmatic reconstruction. According to previous reports, diaphragmatic defects with size ≤5 cm tend to require primary closure by thoracoscopic surgery, and those ≥10 cm tend to require patch repair under thoracotomy.^1^, ^5^ However, there is no consensus on the optimal approach and method due to the rarity of cases.^5^ Therefore, it is important to select the surgical procedure that provides optimal curability and safety in each case.

## CONFLICT OF INTEREST

None declared.

## AUTHOR CONTRIBUTION

Naoya Kitamura drafted and edited this manuscript. Tomohiko Takahashi reviewed the manuscript. Tetsuya Takayama, Jun Kawamukai, Hideki Shinno and Hideki Miyazawa participated in the treatment of the patient and reviewed the manuscript.

## ETHICS STATEMENT

Appropriate written informed consent was obtained for publication of this case report and the images.

## References

[1] Jiang C, Wang H, Chen G, Jiang G, Zhang P. Intradiaphragmatic bronchogenic cyst. Ann Thorac Surg. 2013;96:681–3. 23910110 10.1016/j.athoracsur.2012.10.031

[2] Mubang R, Brady JJ, Mao M. Intradiaphragmatic bronchogenic cysts: case report and systematic review. J Cardiothorac Surg. 2016;11:79. 27150959 10.1186/s13019-016-0444-9 PMC4857253

[3] Herek D, Erbiş H, Kocyigit A, Yagci AB. Retroperitoneal bronchogenic cyst originating from diaphragmatic crura. Indian J Surg. 2015;77:1397–8. 27011575 10.1007/s12262-014-1045-2 PMC4775676

[4] Tokuchi Y, Ukita H, Tsuneta Y, Nishiura Y, Takahashi W, Kawai T, et al. Bronchogenic cyst with high carbohydrate antigen 19‐9 in the cyst fluid and the serum. Intern Med. 1998;37:86–90. 9510408 10.2169/internalmedicine.37.86

[5] Kim JB, Park CK, Kum DY, Lee DH, Jung HR. Bronchogenic cyst of the right hemidiaphragm presenting with pleural effusion. Korean J Thorac Cardiovasc Surg. 2011;44:86–8. 22263133 10.5090/kjtcs.2011.44.1.86 PMC3249282

